# The Role of Platelets in the Stimulation of Neuronal Synaptic Plasticity, Electric Activity, and Oxidative Phosphorylation: Possibilities for New Therapy of Neurodegenerative Diseases

**DOI:** 10.3389/fncel.2021.680126

**Published:** 2021-07-14

**Authors:** Ekaterina Kopeikina, Eugene D. Ponomarev

**Affiliations:** School of Biomedical Sciences, Faculty of Medicine, The Chinese University of Hong Kong, Shatin, Hong Kong

**Keywords:** platelets, neurons, neurodegeneration, major brain gangliosides, serotonin, CNS repair

## Abstract

The central nervous system (CNS) is highly vascularized where neuronal cells are located in proximity to endothelial cells, astroglial limitans, and neuronal processes constituting integrated neurovascular units. In contrast to many other organs, the CNS has a blood-brain barrier (BBB), which becomes compromised due to infection, neuroinflammation, neurodegeneration, traumatic brain injury, and other reasons. BBB disruption is presumably involved in neuronal injury during epilepsy and psychiatric disorders. Therefore, many types of neuropsychological disorders are accompanied by an increase in BBB permeability leading to direct contact of circulating blood cells in the capillaries with neuronal cells in the CNS. The second most abundant type of blood cells are platelets, which come after erythrocytes and outnumber ~100-fold circulating leukocytes. When BBB becomes compromised, platelets swiftly respond to the vascular injury and become engaged in thrombosis and hemostasis. However, more recent studies demonstrated that platelets could also enter CNS parenchyma and directly interact with neuronal cells. Within CNS, platelets become activated by recognizing major brain gangliosides on the surface of astrocytes and neurons and releasing a milieu of pro-inflammatory mediators, neurotrophic factors, and neurotransmitters. Platelet-derived factors directly stimulate neuronal electric and synaptic activity and promote the formation of new synapses and axonal regrowth near the site of damage. Despite such active involvement in response to CNS damage, the role of platelets in neurological disorders was not extensively studied, which will be the focus of this review.

## Introduction

Platelet biology recently gains a particular interest in neuroscience. Quite intriguing that platelet granules, which are organized like vesicles of presynaptic neurons, store key neurotransmitters: dopamine, glutamate, histamine, serotonin, ATP along with proper receptors and transporters for these molecules (Rainesalo et al., 2005). Moreover, platelet granules contain a significant set of neurotrophic factors such as EGF, NGF and, BDNF (Yamamoto and Gurney, 1990; Au et al., 2014; Kniewallner et al., 2014). Finally, platelets and the CNS might be linked via specific structural similarity of platelets with neurons. Both cell types have complex granule organization with diverse content and regulated secretion, as well as the presence of mitochondria and extensive oxidative phosphorylation. Platelet granule secretion and neuronal synaptic release work virtually identically engaging the same triggers and downstream signaling cascades (Ponomarev, 2018). Platelets also contain a variety of pro-inflammatory factors that could strongly influence the pathology of neurodegenerative diseases (Reed et al., 2000). Nevertheless, the platelet role in neuroscience remains neglected. This review aims to elucidate an important role of platelets in most common neurologic disorders: epilepsy, traumatic brain injury, Alzheimer’s disease, multiple sclerosis, and Parkinson’s disease. Future perspectives to use platelets and antiplatelet drugs for diagnostics and treatment of neurologic disorders will be also discussed.

## Epilepsy and Traumatic Brain Injury

Platelets were found in our very recent study (Kopeikina et al., 2020) to actively participate in the development of several epilepsy-associated pathological events in the CNS such as the swift release of serotonin, direct stimulation of neuronal electric activity, blood-brain barrier (BBB) permeabilization, release, and induction of expression of pro-inflammatory mediators, and induction of neuronal oxidative stress (Kopeikina et al., 2020). Previously we found that platelets were also actively involved in TBI pathology (Dukhinova et al., 2018). These findings support the hypothesis that platelets play a significant role in epilepsy development, especially in epilepsy that is associated with TBI.

Epilepsy is a neurological disorder that affects more than 70 million globally and is manifested by the occurrence of seizures due to global or focal abnormally high brain electric activity (Thijs et al., 2019). This disorder is also associated with motor, cognitive, and psychological abnormalities (Devinsky et al., 2018). Although the real cause of epilepsy is unknown, some factors create a predisposition to this disease. Previous studies on epilepsy have mostly focused on genetic factors and pathological events related to neuronal functions in the central nervous system (CNS). Such studies reported that the imbalance between activating and inhibitory circuits in the area of abnormal neuronal electric activity plays a major role in seizure development (Staley, 2015). Blocking inhibitory GABA_A_ receptors with pharmacological antagonists, such as pentylenetetrazole (PTZ), results in seizure development in several species, including humans and mice. The acute PTZ-induced seizure model is widely used to study the effects of new antiepileptic drugs (Löscher, 2011). Stimulation of activating glutamate receptors with specific agonists also results in epileptic seizures (McKhann et al., 2003). Available antiepileptic drugs mostly change the balance toward inhibitory pathways. Yet more than 30% of epilepsy patients do not respond to these drugs (Thijs et al., 2019). Thus, it is critical to find new drugs to treat epilepsy based on other mechanisms and possibly targeting other cell types besides neurons.

Although the main cause for epilepsy is known, a predisposition to this disorder comes from both genetic and environmental factors such as abnormal CNS development and CNS injuries such as stroke and traumatic brain injury (TBI; Devinsky et al., 2018; Thijs et al., 2019). As many as ~50% of all epilepsy cases are triggered by initial neuronal injury and are classified as acquired epilepsy (AE). The three stages of AE include: (1) initial neuronal injury, (2) epileptogenesis, and (3) chronic epilepsy (period of spontaneous recurrent seizures). TBI and stroke are the most frequent brain injuries that often result in the development of AE. AE seizures are classified as acute (hours or days post-TBI) and chronic (from weeks to months). The occurrence of seizures following TBI or other insult is classified as immediate (less than 24 h), early (1–7 days), or late (more than 1 week; Tomkins et al., 2008; Lucke-Wold et al., 2015; Glushakov et al., 2016) AE.

Neuronal functions are substantially influenced by a milieu of platelet-derived factors. One of our key results of whole-genome transcriptome profiling followed by real-time RT PCR validation is that platelets induced expression of many genes related to neuronal electric/synaptic activity, neuroinflammation, and oxidative phosphorylation in TBI and epilepsy models (Dukhinova et al., 2018; Kopeikina et al., 2020). Platelets induced the expression of mRNA for pro-inflammatory cytokines *IL-1B*, *IL-6*, and *TNF* (Kopeikina et al., 2020). The oxidative phosphorylation pathway (e.g., expression of mRNA for mitochondrial genes *MT-CO1*, *MT-ATP6*, and *MT-ND6*) was also significantly upregulated, while the glycolysis pathway is downregulated (Kopeikina et al., 2020). Platelets also upregulated the expression of mRNA for several early response genes responsible for neuronal synaptic plasticity, such as *PSD95, TrkB, Syn1, FOSB*, *EGR1*, *ARC*. We also found that platelets stimulated the formation of dendritic spines and new synapses both *in vitro* and *in vivo* (Dukhinova et al., 2018; Kopeikina et al., 2020).

Serotonin (5HT) is known to contribute to thrombosis, but at the same time, this neurotransmitter is involved in the regulation of innate and adaptive immune responses, neuroinflammation, anaphylaxis, and CNS tissue repair (Sotnikov et al., 2013; Starossom et al., 2015; Dukhinova et al., 2018; Kopeikina et al., 2020). During TBI, we showed that platelet-derived 5HT enhances neuronal axonal growth and the formation of new synapses (Dukhinova et al., 2018). We also demonstrated the significant role of platelets and platelet-derived 5HT in the development of PTZ-induced seizures in mice (Gharedaghi et al., 2014; Carhart-Harris and Nutt, 2017). Our *in vitro* and *in vivo* experiments where we did the co-incubation of platelets with brain slices, or with cultured neurons, or adoptive transfer of serotonin-depleted platelets strongly proved that platelet-derived 5HT was required to increase neuronal electric activity (Dukhinova et al., 2018; Kopeikina et al., 2020).

Quite interesting that in both PTZ-induced epilepsy and TBI models, we observed increased neuronal electric activity, and expression of pro-inflammatory and synaptic plasticity genes (Dukhinova et al., 2018; Kopeikina et al., 2020). Remarkable, intracranial injection of a small volume of platelets, but not saline or platelet-poor plasma, induced severe seizures and epilepsy-like elevated neuronal electrical activity (Kopeikina et al., 2020), which bridge together TBI and epilepsy and may imply the mechanisms of development of acute AE such as post-traumatic or post-stroke epilepsy. An elevated level of oxidative phosphorylation often results in ROS formation leading to oxidative stress in the CNS (Pearson-Smith and Patel, 2017), which was confirmed in our studies where platelets upregulated a large number of mitochondria oxidative phosphorylation genes leading to reactive oxygen species (ROS) formation in neurons leading to neuronal oxidative stress *in vitro* and *in vivo* (Kopeikina et al., 2020).

Thus, we hypothesize that platelets could promote the development of immediate (less than 24 h) AE by directly stimulating neuronal electric activity by robust 5HT release (Kopeikina et al., 2020). Platelets could also stimulate the development of early AE (1–7 days) possibly by upregulation of the number of genes related to neuronal activity and synaptic plasticity, neuroinflammation, and oxidative phosphorylation (Dukhinova et al., 2018; Kopeikina et al., 2020). Finally, platelets could contribute to late AE (more than 1 week) by stimulating axonal regrowth and formation of new synapses in the area around brain injury (Dukhinova et al., 2018), which might lead to the imbalance of excitatory vs. inhibitory circuits (Musto et al., 2016; Pfisterer et al., 2020).

Our studies indicated a new concept that during epilepsy and TBI platelets could enter CNS due to increased BBB permeability and could interact with neuronal cells via cell-cell contacts and/or via secretion of soluble factors. In Table 1, we summarized the role of platelets in epilepsy and TBI. During initial CNS insult such as epileptic seizures or TBI, BBB permeability increases and platelets enter CNS perivascular space where they interact with astroglial and neuronal lipid rafts (Sotnikov et al., 2013) and secrete neurotransmitters (serotonin; Kopeikina et al., 2020), cytokines (IL-1α; Sotnikov et al., 2013; Starossom et al., 2015), chemokines (platelet factor 4, PF4; Starossom et al., 2015) and lipid mediators (platelet-activating factor, PAF; thromboxane, etc.; Dukhinova et al., 2018). At this stage, platelets contribute to the development of neuroinflammation leading to further increase in BBB permeability (Kopeikina et al., 2020) and stimulating macroglia activation and leukocyte infiltration from the periphery (Sotnikov et al., 2013; Starossom et al., 2015; Dukhinova et al., 2018; Ponomarev, 2018). Increased levels of neuroinflammation result in a further increase in BBB permeability. At this stage platelets enter CNS parenchyma and directly interact with neurons by stimulating their electric activity via secretion of serotonin (Kopeikina et al., 2020) and stimulating neuronal gene expression related to synaptic plasticity and oxidative phosphorylation (Dukhinova et al., 2018; Kopeikina et al., 2020). Finally increased expression of neuronal synaptic plasticity genes stimulated the formation of new synapses around the area of injury promoting CNS repair and recovery from the disease (Dukhinova et al., 2018; Table 1). We believe that this concept could be extended to other neurodegenerative diseases, as discussed below.

**Table 1:** Platelet-neural crosstalk and possible mechanisms involved in the development of neurodegenerative diseases and central nervous system (CNS) repair^1^.

Stage of neuro-pathology	Blood-brain barrier (BBB) permeability	Recognition of major brain gangliosides on astroglial and neuronal lipid rafts and secretion of platelet-derived factors	Effect of platelets on CNS function (Role of platelet-derived factors)
Normal CNS	Intact	No	No effect
Primary CNS damage	Mild BBB permeability	Platelets enter CNS perivascular space, recognize major brain gangliosides on astroglial lipid rafts, and secrete serotonin/5-HT, BDNF, PAF, PF4, and IL-1^2, 3^	Platelets enhance BBB permeability (unknown platelet-derived factor)^6^
			Platelets initiate neuroinflammation by stimulating perivascular macrophages and microglia (PAF, PF4, IL-1)^2–^^6^
			Platelets promote blood coagulation (5-HT, ATP)^4, 6^
Secondary CNS damage	Severe BBB permeability	Platelets enter CNS parenchyma, recognize major brain gangliosides on neuronal lipid rafts, and continue to secrete serotonin/5-HT, BDNF, PAF, PF4, and IL-1^2–6^	Platelets stimulate neuronal synaptic activity (5-HT, BDNF)^4, 6^
			Platelets stimulate neuronal electric activity (5-HT)^4, 6^
			Platelets induce mitochondrial oxidative phosphorylation and oxidative stress in neurons (unknown platelet-derived factor)^6^
CNS repair	Decreased BBB permeability	Platelet-derived factors result in marked changes in CNS gene expression profile^4, 6^	Platelets induce expression of neuronal early response genes (5-HT, BDNF)^4, 6^
			Platelets induce the formation of dendritic spines and new synapses (5-HT, BDNF)^4^
			Platelets induce axonal outgrowth (5-HT)^4^

*^1^Proposed scheme based on cited below literature data. ^2^Sotnikov et al. (2013). ^3^Starossom et al. (2015). ^4^Dukhinova et al. (2018). ^5^Ponomarev (2018). ^6^Kopeikina et al. (2020)*.

## Alzheimer’s Disease

It became quite clear after our recent studies that platelets can stimulate neuronal electric and synaptic activity during traumatic brain injury and epilepsy (Dukhinova et al., 2018; Kopeikina et al., 2020). We believe that similar processes of interaction of platelets with neurons could be found in other types of neurological disorders, such as Alzheimer’s disease (AD). AD is a neurodegenerative disorder, which is the common cause of dementia in elderly. Neuropathology includes extracellular β-Amyloid (Aβ) plaques co-localized with ganglioside containing neuronal lipid rafts, neuronal intracellular neurofibrillary tau protein tangles, and neuroinflammation (Masters et al., 2015; Dukhinova et al., 2019). Recent studies demonstrated regulation of BBB permeability by circadian rhythms and sleep (Cuddapah et al., 2019), indicating possibilities for platelet migration into CNS during even early stages of various pathological conditions such as AD. To support this hypothesis, it was shown that Aβ undergoes daily oscillation in interstitial fluid in the vicinity of brain blood vessels, suggesting a possible influx of Aβ from the periphery into CNS (Kress et al., 2018). Very recent studies indicate that platelets can contribute to BBB disruption (Kopeikina et al., 2020; Wu et al., 2021) and can transfer Aβ from blood vessels into CNS (Wu et al., 2021; Table 1). Moreover, it was demonstrated that activated platelets from APP/PS1 transgenic mice invade brain parenchyma and are closely associated with astrocytes (Kniewallner et al., 2020) that are enriched with major brain gangliosides in lipid rafts of astroglial limitations and efficiently activate platelets (Sotnikov et al., 2013). Platelets and their secreted factors could affect many cell types involved in the regulation of BBB integrity including endothelial cells, astroglia, and pericytes (Fang et al., 2011; Gonzales et al., 2020; Kniewallner et al., 2020).

Indeed, outside of CNS, platelets are known as the main source of Aβ (Veitinger et al., 2014; Inyushin et al., 2020). Thus, the cellular mechanisms of AD can be effectively studied using platelet sample preparations, due to dramatically increased levels of the Aβ precursor protein (APP) in them in comparison to all peripheral tissues, and all three isoforms of APP (130, 110, and 106 kDa) being detectable within platelets. During platelet activation, full-length APP is cleaved by a Ca^2+^-dependent cysteine protease, while APP processing is altered in AD patients when compared to healthy individuals. This results in a decreased ratio between the 130 kDa and 106–110 kDa of cleaved APP isoforms, which implies that APP isoform ratios in platelets might act as a biomarker for AD (Tobergte and Curtis, 2013). A recent study demonstrated that Aβ fragments could enter CNS from blood vessels in the mouse model of AD (Bu et al., 2018). Using the parabiosis model, where there was connected the blood system of control (wild-type, WT) mice and transgenic mice with human APP overexpression in the CNS, it was revealed that in a previously healthy WT mouse the brain exhibited signs of AD that include Aβ depositions, neurodegeneration, and neuroinflammation (Bu et al., 2018). Thus, platelets can secrete various processed forms of APP and other substances while infiltrating the brain, which results in the growth of Aβ depositions in the brain and increases the permeability of BBB (Espinosa-Parrilla et al., 2019; Wu et al., 2021). We demonstrated that major brain gangliosides within neuronal lipid rafts in post-synaptic membranes induced platelets’ granule release (Sotnikov et al., 2013) suggesting a possible mechanism of Aβ secretion by platelets in the CNS. At the same time, brain-specific gangliosides serve as an anchor point for binding of Aβ peptides, which was critical for AD development in 5XFAD mouse model (Ponomarev, 2018; Dukhinova et al., 2019).

Besides secretion of Aβ peptides, platelets could also stimulate neuronal electric activity vis production of serotonin (Dukhinova et al., 2018; Kopeikina et al., 2020; Table 1), while neurons with a high level of electric activity were found in close vicinity of Aβ depositions (Busche et al., 2008; Liu et al., 2018). Thus, we could speculate that platelets contribute to elevated neuronal electric activity in AD. The elevated level of electric activity could even result in the development of seizures in some AD patients (Born, 2015; Kitchigina, 2018). Thus, elevated neuronal activity was shown to contribute to AD pathology via multiple mechanisms (Hefter et al., 2020), while our study indicated the critical role of platelets in the stimulation of neurons (Kopeikina et al., 2020).

## Multiple Sclerosis

Altered platelet function is also suggested in autoimmune neuroinflammatory CNS diseases, such as multiple sclerosis (MS), where the level of activated platelets in the blood of patients is elevated (Morel et al., 2017). MS is an autoimmune disease of the nervous system (CNS) that affects predominantly young adults leading to substantial neurological disability that include upper/lower motor syndrome. MS and experimental autoimmune encephalitis (EAE; an animal model for MS) involve autoimmune Th1 and Th17 cells that recognize myelin self-antigen such as MBP, MOG, and PLP. MS onset usually occurs with the relapsing-remitting type (RRMS), which is characterized by multiple relapses followed by spontaneous remission. Platelets were found to aggravate EAE (Langer et al., 2012; Sotnikov et al., 2013) and induce the development of gray matter damage (Sonia D’Souza et al., 2018). During the early stages of EAE platelets entered the hippocampus, and this is linked with the establishment of a neuroinflammatory environment because of platelet-neuron associations, but not with inflammatory cell infiltration, emphasizing the key role of platelets at the preclinical stages of the disease (Kocovski et al., 2019). This effect along with enhanced anxiety-like behavior observed in mice with EAE was ameliorated after the platelet depletion in mice. We have previously shown that platelet-derived 5-HT and PAF (Table 1) boosts the differentiation of pathogenic Th1 and Th17 cells during the early stages of MS and EAE (Ponomarev, 2018), while at later stages of the disease, platelets become depleted in granule content but upregulate adhesion molecules such as CD62P to form aggregates with lymphocytes (Starossom et al., 2015). This, in turn, implies that platelets act as a target to alleviate MS symptoms during relapses, among which are subsequent neuropsychiatric symptoms present in these patients (Kocovski et al., 2019). Targeting platelets would be especially effective during the remission period for prophylaxis of future relapses in RRMS. Thus, platelet-neuronal interaction plays an important role during the early stages of MS/EAE and neuronal dysfunctions, which could especially be important for the prevention of future relapses.

## Parkinson’s Disease

The role of platelets in Parkinson’s disease (PD) is currently unknown. However, there is accumulating knowledge that suggests the possible involvement of platelets in PD pathology. PD is a degenerative disorder caused by the loss of dopaminergic neurons in the substantia nigra, which leads to a decrease in motor and cognitive functions. There is no final answer to what causes PD, though mitochondrial dysfunction is implied to be one of the major reasons. A toxin 1-methyl-4-phenyl-1,2,3,6-tetrahydropyridine (MPTP), which is selectively toxic for dopaminergic neurons, was found to act by inhibiting complex I of the electron transport chain in neuronal mitochondria, which is important for oxidative phosphorylation. It is known that MPTP is widely used as a chemical intermediate for herbicide production, that could contaminate agricultural products and predispose them to PD (Fukuda, 2001). In addition to herbicides, it was found that insecticide rotenone is also toxic for neurons in the substantia nigra affecting complex I in neuronal mitochondria leading to the symptoms of PD (Sherer et al., 2003).

Decreased complex I activity was found in the lymphocytes and platelets isolated from PD patients when compared to healthy controls (Haas et al., 1995; Subrahmanian and LaVoie, 2021). Moreover, a hybrid PD model where mitochondrial DNA from PD platelets was expressed in human teratocarcinoma cells demonstrated decreased complex I activity in mitochondria of these cells. Besides, 1-methyl-4-phenyl-pyridinium ion (MPP^+^), the metabolite of MPTP, was proved to deplete adenosine triphosphate in platelets and induce attenuated platelet aggregation and activity, which is a probable mechanism of the anti-aggregation effect found in PD patients (Koçer et al., 2013; Leiter and Walker, 2020). Monoamine oxidase B (MAO-B) enzyme, which is abundant in neuronal and platelet mitochondria, also contributes significantly to MPTP toxicity and the etiology of PD as demonstrated by several studies. Enhanced MAO-B activity has been discovered in PD patients, nevertheless, the findings regarding platelet MAO-B activity in PD patients are not straightforward enough, as other works imply that platelet MAO-B activity is unaffected in PD patients (Bonuccelli et al., 1990). Nevertheless, the ability of platelets to modulate the functions of mitochondria in the CNS by enhancing oxidative phosphorylation and oxidative stress (Kopeikina et al., 2020) could play an important role in PD pathology.

Besides modulation of neuronal mitochondrial functions by platelets, these cells could affect dopaminergic neurons by producing several substances. Among these factors are a platelet-activating factor (PAF; Table 1), which is produced by activated platelets and plays a pro-inflammatory role in the development of TBI-associated neuroinflammation affecting astrocytes and microglia (Yin et al., 2017; Dukhinova et al., 2018). In addition to glial cells, the PAF receptor (PAFR) is also expressed in neuronal synapses. Stimulation of neurons via PAFR enhanced long-term potentiation and synaptic vesicle release (Hammond et al., 2016). On the other hand, overstimulation of neurons by PAF caused neuronal apoptosis, although the role of PAFR in this process remains controversial (Bennett et al., 1998; Ryan et al., 2007). Mice deficient in PAFR were found to be resistant to the development of disease in a mouse model of MPTP-induced PD. Thus, platelet-derived factors such as PAF could significantly contribute to the development of PD.

## Platelets as Markers for Diagnostics

Recently, platelets have come to be regarded as substantial indicators for neurologic diseases of various types. Being multifunctional blood anucleated cells, they are now seen as crucial clinical targets for many neurologic disease pathophysiology. Not only do platelets play a key role in normal hemostasis and thrombosis, but they also are first responders in inflammatory processes (Sotnikov et al., 2013; Starossom et al., 2015; Ponomarev, 2018). They are also involved in a vast range of inflammation-associated pathologies, such as atherosclerosis, cardiovascular diseases, cancer metastasis, and neurodegenerative disorders (Koçer et al., 2013; Franco et al., 2015; Pluta et al., 2018).

During many types of neurodegenerative diseases such as TBI, AD, and MS, platelets change their phenotype and content (Kumar, 2013; Koc et al., 2014; Starossom et al., 2015; Ponomarev, 2018). For example, during AD, platelets show an activated phenotype and there are changes in the processing of platelet Aβ, which could serve as an early marker of AD (Di Luca et al., 1998; Koc et al., 2014). In the mouse model of AD when human APP with Swedish mutation is expressed in the CNS, platelets start to express MMP2 and MMP-9 and damage brain blood vessels in the brain slice model (Kniewallner et al., 2018). This data indicates that CNS-derived amyloid could activate platelets that could contribute to increased BBB permeability. Our data in the epilepsy model also demonstrated that platelets contribute to increased BBB permeability (Kopeikina et al., 2020). In MS, platelets decrease their level of serotonin in dense granules and the level of PF4 in α-granules and upregulate adhesion molecules such as CD62P (Starossom et al., 2015). Quite interestingly the exhaustion of platelet granule content and upregulation of CD62P make platelets anti-inflammatory indicating that platelets play a differential role in the early and late stages of MS (Starossom et al., 2015). This indicates the importance of looking at various parameters to characterize detrimental and beneficial phenotypes of activated platelets to target pathogenic platelet subsets. Imaging cytometry has great potential to assess platelet content such as microRNA (Ponomarev et al., 2011). However, currently, it is challenging, especially in the case of neurologic disorders.

**Figure 1:**
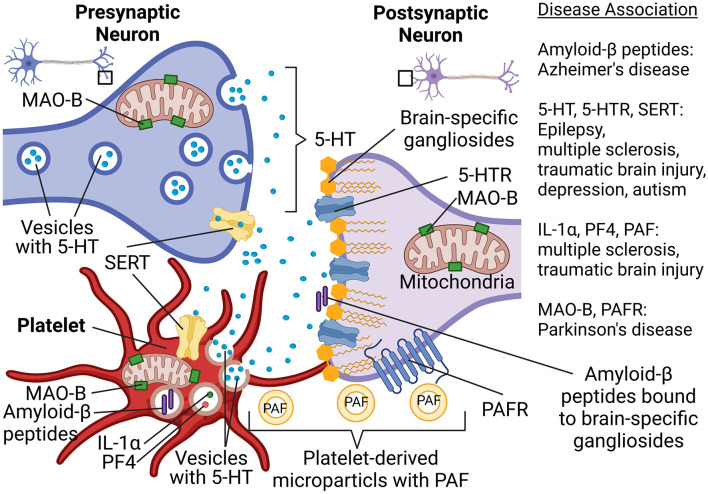
Platelet-neuron similarity and platelet-neuron interactions have implications for a wide range of neuropsychological disorders such as Alzheimer’s disease, Parkinson’s disease, multiple sclerosis, traumatic brain epilepsy, and depression. Abbreviations: 5-HT, Serotonin; 5-HTR, 5-HT receptor; IL-1α, Interleukin 1α; MAO-B, Monoamine oxidase B; PAF, Platelet-activating factor; PAFR, PAF receptor; PF4, Platelet factor-4; SERT, Serotonin transporter. Created in BioRender.com.

To better understand the possible involvement of platelets in neurologic disorders, a new concept was proposed by several scientists several years ago (Ponomarev, 2018). According to this concept, platelets can be viewed as “circulating mirrors” of neurons and innate immune cells (Ponomarev, 2018; Canobbio, 2019; Figure 1). In the coming years, the analysis of platelet morphology, functionality, metabolism, as well as protein and lipid composition may advance the investigation of neurodegenerative diseases (Figure 1). For example, analysis of metabolism and secretion of serotonin could be used for the understanding of predisposition and progression of depression and TBI. Platelets also use serotonin transporter (SERT), which is targeted by selective serotonin reuptake inhibitors, and anti-depressant drugs (Figure 1). Moreover, we recently demonstrated an important role of serotonin in epilepsy and the formation of new synapses. The latter also plays a role in the development of autism spectrum disorder. The ability of platelets to store, process, and secrete various forms of Aβ could be used as a marker for AD. Analysis of platelets’ mitochondrial function could be used to diagnose predisposition for PD (Figure 1). Thus, platelets can become a suitable tool for the analysis of peripheral biomarkers when it comes to diagnosing neuronal dysfunctions in the future (Ehrlich, 2012; Oji et al., 2018; Canobbio, 2019; Padmakumar et al., 2019).

## Platelets as Targets for Future Therapy

Targeting platelets and their products for therapeutic purposes in the field of neurological pathologies seems to present an exceptional challenge, both because of the blood-brain barrier and due to obstacles for the drugs on their way to the desired locations of action inside the CNS parenchyma. Above all else, there might be demand for various approaches on how to target platelet or platelet-derived microparticles in circulation and CNS parenchyma in the same patients. These interventions will require advanced drug formulations and delivery techniques, along with modern imaging methods to determine drug stability, accurate targeting, and efficacy. Antiplatelet therapy is broadly applied in the treatment and prevention of patients with thrombotic cerebrovascular or cardiovascular diseases. Platelet aggregation and activation can be decreased by antiplatelet therapy with one or several drugs available on the market with the most common being heparin and aspirin (Gianazza et al., 2020). There are several antiplatelet drugs currently available on the market with the most popular being aspirin (Chandrasekhara et al., 2016). In the 1960s aspirin appeared as the first valid antiplatelet drug and is still applied to prevent cardiovascular pathologies.

Aspirin was found to ameliorate spontaneous recurrent seizures in SWR/J mice with pilocarpine-induced chronic temporal lobe epilepsy, however, the role of platelets was not investigated in this study (Zhu et al., 2017). It was an interesting case study documenting that woman with epilepsy history who constantly took aspirin (75 mg daily for 3 years) had epileptic convulsion 3 weeks after aspirin withdrawal, but not during the period when she took aspirin (Scheepers et al., 2007). A preliminary clinical study also showed decreased seizure frequency for epilepsy patients on day 2 after starting to take aspirin. The authors of this study emphasize that further prospective study is needed towards this direction (Godfred et al., 2013). Thus, the usage of aspirin (and possibly other antiplatelet drugs) is a perspective for the prevention and treatment of epilepsy. At present, the main platelet target which is used as a target in antiplatelet therapy is cyclooxygenase-1/2 (COX-1/2) with more selective targeting of COX-1. COX-1 is responsible for regulating the production of the prostaglandins, which control platelet aggregation and activation, and is affected by irreversible cyclooxygenase inhibitors such as NSAIDs. NSAIDs were found to be promising for prophylaxis of AD; however, these drugs decrease neuroinflammation by targeting endothelial cells and macrophages and it is not entirely platelet specific (Zhang et al., 2018). Another currently approved more specific antiplatelet drug clopidogrel or its analogs (P2Y_12_ inhibitors) target ADP receptors on platelets significantly reducing their activation (Amin et al., 2017). Usage of this drug for the prevention and treatment of neurodegenerative diseases is very limited; however, it was recently reported that clopidogrel reduced pathology in the rat model of AD (Khalaf et al., 2020). New drugs and their platelet targets are still being studied, and some promising targets are at the stage of clinical investigation to discover new possible biomarkers for accurate treatment (Coppinger et al., 2007; Yousuf and Bhatt, 2011; Dovizio et al., 2014).

Knowing the important role of platelets in neurodegenerative diseases, it is time to rethink the mechanism of action of some of the known drugs widely used for the treatment of neurologic conditions. There are several candidates for re-evaluation of their therapeutic mechanisms. A common drug for MS called glatiramer acetate (Copaxone^TM^) in addition to targeting T cells also targets platelets inhibiting their calcium influx, activation, aggregation, and prolongs bleeding time (Starossom et al., 2014). valproic acid, a common drug for the treatment of epilepsy and bipolar disorder, could also affect platelet functions by decreasing their numbers causing thrombocytopenia. So these two drugs could be also called anti-platelet agents.

Most current platelet-specific drugs non-specifically inhibit platelet activation and aggregation, which may cause dangerous complications such as bleeding. Moreover, in certain neurodegenerative diseases, platelets’ granule content becomes exhausted and their ability to get further activated is decreased (Starossom et al., 2015), which may require platelet functions to be restored and pathogenic platelets to be replaced by non-pathogenic platelets. To do so, there is a possibility of using autologous genetically engineered iPSC-derived platelets, or artificial platelet-like microparticles for transfusion of patients with neurological disorders (Brown et al., 2014; Moreau et al., 2016; Lawrence et al., 2019). Yet, a more detailed investigation of detrimental vs. beneficial pathways of platelet activation during neurodegenerative diseases should be performed to understand specific pathways in platelets to be targeted using these novel technologies.

## Concluding Remarks

Recent research results demonstrated that platelets play a crucial role in the pathology of multiple neurologic disorders. During many types of neurodegenerative diseases, the BBB is compromised, which enables platelets to infiltrate the central nervous system (CNS), where they release serotonin and other mediators. Enhanced neuronal electric activity and seizure severity have been greatly contributed to by platelet-derived (but CNS-derived) serotonin and possibly other platelet-derived co-factors. Besides, a whole-genome transcriptome analysis suggested that platelets trigger the expression of many genes associated with neuronal synaptic activity, neuroinflammation, and oxidative phosphorylation in neuronal mitochondria. Based on this, we introduced the model shown in Table 1. Our model implies that platelets could provide the “missing link” between TBI and epilepsy, or initial neuronal damage and further development of other neurodegenerative pathologies such as AD, PD, and MS (Figure 1). Thus, the main conclusion of our review is that platelets cannot be any further ignored in modern neurobiology.

## Author Contributions

EK and EP conceived and wrote the manuscript. EP edited the manuscript. EK made Figure 1. EP made Table 1. All authors contributed to the article and approved the submitted version.

## Conflict of Interest

The authors declare that the research was conducted in the absence of any commercial or financial relationships that could be construed as a potential conflict of interest.

## References

[B1] Amin A. M. Sheau Chin L. Azri Mohamed Noor D. Sk Abdul Kader M. A. Kah Hay Y. Ibrahim B.. (2017). The personalization of clopidogrel antiplatelet therapy: the role of integrative pharmacogenetics and pharmacometabolomics. Cardiol. Res. Pract. 2017:8062796. 10.1155/2017/8062796 28421156 PMC5379098

[B2] Au A. E. L. Sashindranath M. Borg R. J. Kleifeld O. Andrews R. K. Gardiner E. E.. (2014). Activated platelets rescue apoptotic cells *via* paracrine activation of EGFR and DNA-dependent protein kinase. Cell Death Dis. 5:e1410. 10.1038/cddis.2014.373 25210793 PMC4540201

[B3] Bennett S. A. L. Chen J. Pappas B. A. Roberts D. C. S. Tenniswood M. (1998). Platelet activating factor receptor expression is associated with neuronal apoptosis in an *in vivo* model of excitotoxicity. Cell Death Differ. 5, 867–875. 10.1038/sj.cdd.4400434 10203694

[B4] Bonuccelli U. Piccini P. Del Dotto P. Muratorio A. Pacifici G. M. Corsini G. U. (1990). Platelet monoamine oxidase B activity in Parkinsonian patients. J. Neurol. Neurosurg. Psychiatry 53, 854–855. 10.1136/jnnp.53.10.854 2266365 PMC488245

[B5] Born H. A. (2015). Seizures in Alzheimer’s disease. Neuroscience 286, 251–263. 10.1016/j.neuroscience.2014.11.051 25484360

[B6] Brown A. C. Stabenfeldt S. E. Ahn B. Hannan R. T. Dhada K. S. Herman E. S.. (2014). Ultrasoft microgels displaying emergent platelet-like behaviours. Nat. Mater. 13, 1108–1114. 10.1038/nmat4066 25194701 PMC4239187

[B7] Bu X. L. Xiang Y. Jin W. S. Wang J. Shen L. L. Huang Z. L.. (2018). Blood-derived amyloid-β protein induces Alzheimer’s disease pathologies. Mol. Psychiatry 23, 1948–1956. 10.1038/mp.2017.204 29086767

[B8] Busche M. A. Eichhoff G. Adelsberger H. Abramowski D. Wiederhold K. H. Haass C.. (2008). Clusters of hyperactive neurons near amyloid plaques in a mouse model of Alzheimer’s disease. Science 321, 1686–1689. 10.1126/science.1162844 18802001

[B9] Canobbio I. (2019). Blood platelets: circulating mirrors of neurons? Res. Pract. Thromb. Haemost. 3, 564–565. 10.1002/rth2.12254 31624775 PMC6781913

[B11] Carhart-Harris R. L. Nutt D. J. (2017). Serotonin and brain function: a tale of two receptors. J. Psychopharmacol. 31, 1091–1120. 10.1177/0269881117725915 28858536 PMC5606297

[B12] Chandrasekhara V. Chathadi K V. Early D. S. Eloubeidi M. A. Evans J. A. Faulx A. L.. (2016). The management of antithrombotic agents for patients undergoing GI endoscopy. Gastrointest. Endosc. 83, 3–16. 10.1016/j.gie.2015.09.035 26621548

[B13] Coppinger J. A. O’Connor R. Wynne K. Flanagan M. Sullivan M. Maguire P. B.. (2007). Moderation of the platelet releasate response by aspirin. Blood 109, 4786–4792. 10.1182/blood-2006-07-038539 17303692

[B14] Cuddapah V. A. Zhang S. L. Sehgal A. (2019). Regulation of the blood-brain barrier by circadian rhythms and sleep. Trends Neurosci. 42, 500–510. 10.1016/j.tins.2019.05.001 31253251 PMC6602072

[B15] Devinsky O. Vezzani A. O’Brien T. J. Jette N. Scheffer I. E. De Curtis M.. (2018). Epilepsy. Nat. Rev. Dis. Primers 4:18024. 10.1038/nrdp.2018.24 29722352

[B16] Di Luca M. Pastorino L. Bianchetti A. Perez J. Vignolo L. A. Lenzi G. L.. (1998). Differential level of platelet amyloid β precursor protein isoforms: an early marker for Alzheimer disease. Arch. Neurol. 55, 1195–1200. 10.1001/archneur.55.9.1195 9740113

[B17] Dovizio M. Alberti S. Guillem-Llobat P. Patrignani P. (2014). Role of platelets in inflammation and cancer: novel therapeutic strategies. Basic Clin. Pharmacol. Toxicol. 114, 118–127. 10.1111/bcpt.12156 24118902

[B18] Dukhinova M. Kuznetsova I. Kopeikina E. Veniaminova E. Yung A. W. Y. Veremeyko T.. (2018). Platelets mediate protective neuroinflammation and promote neuronal plasticity at the site of neuronal injury. Brain Behav. Immun. 74, 7–27. 10.1016/j.bbi.2018.09.009 30217533

[B19] Dukhinova M. Veremeyko T. Amanda W. Y. Y. Kuznetsova I. N. S. Lau T. Y. B. Kopeikina E.. (2019). Fresh evidence for major brain gangliosides as a target for the treatment of Alzheimer’s disease. Neurobiol. Aging 77, 128–143. 10.1016/j.neurobiolaging.2019.01.020 30797170

[B20] Ehrlich D. (2012). Platelets in psychiatric disorders. World J. Psychiatry 2:91. 10.5498/wjp.v2.i6.91 24175174 PMC3782188

[B21] Espinosa-Parrilla Y. Gonzalez-Billault C. Fuentes E. Palomo I. Alarcón M. (2019). Decoding the role of platelets and related microRNAs in aging and neurodegenerative disorders. Front. Aging Neurosci. 10:151. 10.3389/fnagi.2019.00151 31312134 PMC6614495

[B22] Fang W. Geng X. Deng Y. Li Y. Shang E. Cen J.. (2011). Platelet activating factor induces blood brain barrier permeability alteration in vitro. J. Neuroimmunol. 230, 42–47. 10.1016/j.jneuroim.2010.08.015 20870297

[B23] Franco A. T. Corken A. Ware J. (2015). Platelets at the interface of thrombosis, inflammation and cancer. Blood 126, 582–588. 10.1182/blood-2014-08-531582 26109205 PMC4520875

[B24] Fukuda T. (2001). Neurotoxicity of MPTP. Neuropathology 21, 323–332. 10.1046/j.1440-1789.2001.00402.x 11837540

[B25] Gharedaghi M. H. Seyedabadi M. Ghia J. E. Dehpour A. R. Rahimian R. (2014). The role of different serotonin receptor subtypes in seizure susceptibility. Exp. Brain Res. 232, 347–367. 10.1007/s00221-013-3757-0 24232860

[B26] Gianazza E. Brioschi M. Baetta R. Mallia A. Banfi C. (2020). Platelets in healthy and disease states: from biomarkers discovery to drug targets identification by proteomics. Int. J. Mol. Sci. 21:4541. 10.3390/ijms21124541 32630608 PMC7352998

[B27] Glushakov A. V. Glushakova O. Y. Doré S. Carney P. R. Hayes R. L. (2016). “Animal models of posttraumatic seizures and epilepsy,” in Methods in Molecular Biology, (Totowa, NJ: Humana Press Inc.), 481–519. 10.1007/978-1-4939-3816-2_27 PMC6036905 27604735

[B28] Godfred R. M. Parikh M. S. Haltiner A. M. Caylor L. M. Sepkuty J. P. Doherty M. J.. (2013). Does aspirin use make it harder to collect seizures during elective video-EEG telemetry? Epilepsy Behav. 27, 115–117. 10.1016/j.yebeh.2012.12.031 23399946

[B29] Gonzales A. L. Klug N. R. Moshkforoush A. Lee J. C. Lee F. K. Shui B.. (2020). Contractile pericytes determine the direction of blood flow at capillary junctions. Proc. Natl. Acad. Sci. U S A 117, 27022–27033. 10.1073/pnas.1922755117 33051294 PMC7604512

[B30] Haas R. H. Nasirian F. Nakano K. Ward D. Pay M. Hill R.. (1995). Low platelet mitochondrial complex I and complex II/III activity in early untreated parkinson’s disease. Ann. Neurol. 37, 714–722. 10.1002/ana.410370604 7778844

[B31] Hammond J. W. Lu S.-M. Gelbard H. A. (2016). Platelet activating factor enhances synaptic vesicle exocytosis *via* pkc, elevated intracellular calcium and modulation of synapsin 1 dynamics and phosphorylation. Front. Cell. Neurosci. 9:505. 10.3389/fncel.2015.00505 26778968 PMC4705275

[B32] Hefter D. Ludewig S. Draguhn A. Korte M. (2020). Amyloid, APP and electrical activity of the brain. Neuroscientist 26, 231–251. 10.1177/1073858419882619 31779518 PMC7222965

[B33] Inyushin M. Zayas-Santiago A. Rojas L. Kucheryavykh L. (2020). On the role of platelet-generated amyloid beta peptides in certain amyloidosis health complications. Front. Immunol. 11:2587. 10.3389/fimmu.2020.571083 33123145 PMC7567018

[B34] Khalaf N. E. A. El Banna F. M. Youssef M. Y. Mosaad Y. M. Daba M. H. Y. Ashour R. H. (2020). Clopidogrel combats neuroinflammation and enhances learning behavior and memory in a rat model of Alzheimer’s disease. Pharmacol. Biochem. Behav. 195:172956. 10.1016/j.pbb.2020.172956 32474163

[B35] Kitchigina V. F. (2018). Alterations of coherent theta and gamma network oscillations as an early biomarker of temporal lobe epilepsy and Alzheimer’s disease. Front. Integr. Neurosci. 12:36. 10.3389/fnint.2018.00036 30210311 PMC6119809

[B36] Kniewallner K. M. de Sousa D. M. B. Unger M. S. Mrowetz H. Aigner L. (2020). Platelets in amyloidogenic mice are activated and invade the brain. Front. Neurosci. 14:129. 10.3389/fnins.2020.00129 32194368 PMC7063083

[B37] Kniewallner K. M. Foidl B. M. Humpel C. (2018). Platelets isolated from an Alzheimer mouse damage healthy cortical vessels and cause inflammation in an organotypic *ex vivo* brain slice model. Sci. Rep. 8, 1–16. 10.1038/s41598-018-33768-2 30341392 PMC6195547

[B38] Kniewallner K. M. Grimm N. Humpel C. (2014). Platelet-derived nerve growth factor supports the survival of cholinergic neurons in organotypic rat brain slices. Neurosci. Lett. 574, 64–69. 10.1016/j.neulet.2014.05.033 24861506 PMC4311057

[B39] Koçer A. Yaman A. Niftaliyev E. Dürüyen H. Eryilmaz M. Koçer E.. (2013). Assessment of platelet indices in patients with neurodegenerative diseases: mean platelet volume was increased in patients with Parkinson’s disease. Curr. Gerontol. Geriatr. Res. 2013:986254. 10.1155/2013/986254 24382959 PMC3870626

[B40] Koc E. R. Uzar E. Cirak Y. Demir Y. P. Ilhan A. (2014). The increase of mean platelet volume in patients with Alzheimer disease. Turk. J. Med. Sci. 44:1060. 10.3906/sag-1212-5 25552162

[B41] Kocovski P. Jiang X. D’Souza C. Li Z. Dang P. Wang X.. (2019). Platelet depletion is effective in ameliorating anxiety-like behavior and reducing the pro-inflammatory environment in the hippocampus in murine experimental autoimmune encephalomyelitis. J. Clin. Med. 8:162. 10.3390/jcm8020162 30717130 PMC6406682

[B42] Kopeikina E. Dukhinova M. Yung A. W. Y. Veremeyko T. Kuznetsova I. S. Lau T. Y. B.. (2020). Platelets promote epileptic seizures by modulating brain serotonin level, enhancing neuronal electric activity and contributing to neuroinflammation and oxidative stress. Prog. Neurobiol. 188:101783. 10.1016/j.pneurobio.2020.101783 32142857

[B43] Kress G. J. Liao F. Dimitry J. Cedeno M. R. FitzGerald G. A. Holtzman D. M.. (2018). Regulation of amyloid-β dynamics and pathology by the circadian clock. J. Exp. Med. 215, 1059–1068. 10.1084/jem.20172347 29382695 PMC5881473

[B44] Kumar M. A. (2013). Coagulopathy associated with traumatic brain injury. Curr. Neurol. Neurosci. Rep. 13:391. 10.1007/s11910-013-0391-y 24046182

[B45] Löscher W. (2011). Critical review of current animal models of seizures and epilepsy used in the discovery and development of new antiepileptic drugs. Seizure 20, 359–368. 10.1016/j.seizure.2011.01.003 21292505

[B46] Langer H. F. Choi E. Y. Zhou H. Schleicher R. Chung K. J. Tang Z.. (2012). Platelets contribute to the pathogenesis of experimental autoimmune encephalomyelitis. Circ. Res. 110, 1202–1210. 10.1161/CIRCRESAHA.111.256370 22456181 PMC3382058

[B47] Lawrence M. Mueller A. Ghevaert C. (2019). Using genome editing to engineer universal platelets. Emerg. Top. Life Sci. 3, 301–311. 10.1042/ETLS20180153 33523140 PMC7289015

[B48] Leiter O. Walker T. L. (2020). Platelets in neurodegenerative conditions—friend or foe? Front. Immunol. 11:747. 10.3389/fimmu.2020.00747 32431701 PMC7214916

[B49] Liu D. Lu H. Stein E. Zhou Z. Yang Y. Mattson M. P. (2018). Brain regional synchronous activity predicts tauopathy in 3×TgAD mice. Neurobiol. Aging 70, 160–169. 10.1016/j.neurobiolaging.2018.06.016 30015035 PMC6196326

[B50] Lucke-Wold B. P. Nguyen L. Turner R. C. Logsdon A. F. Chen Y. W. Smith K. E.. (2015). Traumatic brain injury and epilepsy: Underlying mechanisms leading to seizure. Seizure 33, 13–23. 10.1016/j.seizure.2015.10.002 26519659 PMC12767287

[B51] Masters C. L. Bateman R. Blennow K. Rowe C. C. Sperling R. A. Cummings J. L. (2015). Alzheimer’s disease. Nat. Rev. Dis. Primers 1:15056. 10.1038/nrdp.2015.56 27188934

[B52] McKhann G. M. Wenzel H. J. Robbins C. A. Sosunov A. A. Schwartzkroin P. A. (2003). Mouse strain differences in kainic acid sensitivity, seizure behavior, mortality and hippocampal pathology. Neuroscience 122, 551–561. 10.1016/s0306-4522(03)00562-1 14614919

[B53] Moreau T. Evans A. L. Vasquez L. Tijssen M. R. Yan Y. Trotter M. W.. (2016). Large-scale production of megakaryocytes from human pluripotent stem cells by chemically defined forward programming. Nat. Commun. 7:11208. 10.1038/ncomms11208 27052461 PMC4829662

[B54] Morel A. Rywaniak J. Bijak M. Miller E. Niwald M. Saluk J. (2017). Flow cytometric analysis reveals the high levels of platelet activation parameters in circulation of multiple sclerosis patients. Mol. Cell Biochem. 430, 69–80. 10.1007/s11010-017-2955-7 28210898 PMC5437150

[B55] Musto A. E. Rosencrans R. F. Walker C. P. Bhattacharjee S. Raulji C. M. Belayev L.. (2016). Dysfunctional epileptic neuronal circuits and dysmorphic dendritic spines are mitigated by platelet-activating factor receptor antagonism. Sci. Rep. 6, 1–16. 10.1038/srep30298 27444269 PMC4957208

[B56] Oji S. Tomohisa D. Hara W. Tajima T. Suzuki M. Saito A.. (2018). Mean platelet volume is associated with early neurological deterioration in patients with branch atheromatous disease: involvement of platelet activation. J. Stroke Cerebrovasc. Dis. 27, 1–8. 10.1016/j.jstrokecerebrovasdis.2018.01.012 29428328

[B57] Padmakumar M. Van Raes E. Van Geet C. Freson K. (2019). Blood platelet research in autism spectrum disorders: in search of biomarkers. Res. Pract. Thromb. Haemost. 3, 566–577. 10.1002/rth2.12239 31624776 PMC6781926

[B58] Pearson-Smith J. N. Patel M. (2017). Metabolic dysfunction and oxidative stress in epilepsy. Int. J. Mol. Sci. 18:2365. 10.3390/ijms18112365 29117123 PMC5713334

[B59] Pfisterer U. Petukhov V. Demharter S. Meichsner J. Thompson J. J. Batiuk M. Y.. (2020). Identification of epilepsy-associated neuronal subtypes and gene expression underlying epileptogenesis. Nat. Commun. 11, 1–19. 10.1038/s41467-020-18752-7 33028830 PMC7541486

[B60] Pluta R. Ułamek-Kozioł M. Januszewski S. Czuczwar S. J. (2018). Platelets, lymphocytes and erythrocytes from Alzheimer’s disease patients: the quest for blood cell-based biomarkers. Folia Neuropathol. 56, 14–20. 10.5114/fn.2018.74655 29663736

[B61] Ponomarev E. D. (2018). Fresh evidence for platelets as neuronal and innate immune cells: their role in the activation, differentiation and deactivation of Th1, Th17 and tregs during tissue inflammation. Front. Immunol. 9:406. 10.3389/fimmu.2018.00406 29599771 PMC5863511

[B62] Ponomarev E. D. Veremeyko T. Barteneva N. S. (2011). Visualization and quantitation of the expression of microRNAs and their target genes in neuroblastoma single cells using imaging cytometry. BMC Res. Notes 4:517. 10.1186/1756-0500-4-517 22123030 PMC3250958

[B63] Rainesalo S. Keränen T. Saransaari P. Honkaniemi J. (2005). GABA and glutamate transporters are expressed in human platelets. Mol. Brain Res. 141, 161–165. 10.1016/j.molbrainres.2005.08.013 16198020

[B64] Reed G. L. Fitzgerald M. L. Polgar J. (2000). Molecular mechanisms of platelet exocytosis: Insights into the “secrete” life of thrombocytes. Blood 96, 3334–3342. 10.1182/blood.V96.10.3334 11071625

[B65] Ryan S. D. Harris C. S. Mo F. Lee H. Hou S. T. Bazan N. G.. (2007). Platelet activating factor-induced neuronal apoptosis is initiated independently of its G-protein coupled PAF receptor and is inhibited by the benzoate orsellinic acid. J. Neurochem. 103, 88–97. 10.1111/j.1471-4159.2007.04740.x 17877634

[B66] Scheepers M. Pearson A. Michaelides M. (2007). Epileptic convulsion following aspirin withdrawal before lid surgery. Eye 21:446. 10.1038/sj.eye.6702618 17041574

[B67] Sherer T. B. Betarbet R. Testa C. M. Seo B. B. Richardson J. R. Kim J. H.. (2003). Mechanism of toxicity in rotenone models of Parkinson’s disease. J. Neurosci. 23, 10756–10764. 10.1523/JNEUROSCI.23-34-10756.2003 14645467 PMC6740985

[B68] Sonia D’Souza C. Li Z. Luke Maxwell D. Trusler O. Murphy M. Crewther S.. (2018). Platelets drive inflammation and target gray matter and the retina in autoimmune-mediated encephalomyelitis. J Neuropathol. Exp. Neurol. 77, 567–576. 10.1016/j.ymthe.2021.01.033 29757405

[B69] Sotnikov I. Veremeyko T. Starossom S. C. Barteneva N. Weiner H. L. Ponomarev E. D. (2013). Platelets recognize brain-specific glycolipid structures, respond to neurovascular damage and promote neuroinflammation. PLoS One 8:e58979. 10.1371/journal.pone.0058979 23555611 PMC3608633

[B70] Staley K. (2015). Molecular mechanisms of epilepsy. Nat. Neurosci. 18, 367–372. 10.1038/nn.3947 25710839 PMC4409128

[B71] Starossom S. C. Veremeyko T. Dukhinova M. Yung A. W. Y. Ponomarev E. D. (2014). Glatiramer acetate (Copaxone) modulates platelet activation and inhibits thrombin-induced calcium influx: possible role of copaxone in targeting platelets during autoimmune neuroinflammation. PLoS One 9:e96256. 10.1371/journal.pone.0096256 24788965 PMC4008572

[B72] Starossom S. C. Veremeyko T. Yung A. W. Y. Dukhinova M. Au C. Lau A. Y.. (2015). Platelets play differential role during the initiation and progression of autoimmune neuroinflammation. Circ. Res. 117, 779–792. 10.1161/CIRCRESAHA.115.306847 26294656 PMC4716010

[B73] Subrahmanian N. LaVoie M. J. (2021). Is there a special relationship between complex I activity and nigral neuronal loss in Parkinson’s disease? a critical reappraisal. Brain Res. 2021:147434. 10.1016/j.brainres.2021.147434 33745923 PMC9520341

[B74] Thijs R. D. Surges R. O’Brien T. J. Sander J. W. (2019). Epilepsy in adults. Lancet 393, 689–701. 10.1016/S0140-6736(18)32596-0 30686584

[B75] Tobergte D. R. Curtis S. (2013). Neuropsychiatric disorders. An integrative approach. J. Chem. Inf. Model 53, 1689–1699.23800267

[B76] Tomkins O. Shelef I. Kaizerman I. Eliushin A. Afawi Z. Misk A.. (2008). Blood-brain barrier disruption in post-traumatic epilepsy. J. Neurol. Neurosurg. Psychiatry 79, 774–777. 10.1136/jnnp.2007.126425 17991703

[B77] Veitinger M. Varga B. Guterres S. B. Zellner M. (2014). Platelets, a reliable source for peripheral Alzheimer’s disease biomarkers? Acta Neuropathol. Commun. 2:65. 10.1186/2051-5960-2-65 24934666 PMC4229876

[B78] Wu T. Chen L. Zhou L. Xu J. Guo K. (2021). Platelets transport β-amyloid from the peripheral blood into the brain by destroying the blood-brain barrier to accelerate the process of Alzheimer’s disease in mouse models. Aging (Albany NY) 13, 7644–7659. 10.18632/aging.202662 33668038 PMC7993748

[B79] Yamamoto H. Gurney M. E. (1990). Human platelets contain brain-derived neurotrophic factor. J. Neurosci. 10, 3469–3478. 10.1523/JNEUROSCI.10-11-03469.1990 2230938 PMC6570101

[B80] Yin X. J. Chen Z. Y. Zhu X. N. Hu J. J. (2017). Loss of PAFR prevents neuroinflammation and brain dysfunction after traumatic brain injury. Sci. Rep. 7, 1–12. 10.1038/srep40614 28094295 PMC5240097

[B81] Yousuf O. Bhatt D. L. (2011). The evolution of antiplatelet therapy in cardiovascular disease. Nat. Rev. Cardiol. 8, 547–559. 10.1038/nrcardio.2011.96 21750497

[B82] Zhang C. Wang Y. Wang D. Zhang J. Zhang F. (2018). NSAID exposure and risk of Alzheimer’s disease: an updated meta-analysis from cohort studies. Front. Aging Neurosci. 10:83. 10.3389/fnagi.2018.00083 29643804 PMC5882872

[B83] Zhu K. Hu M. Yuan B. Liu J. X. Liu Y. (2017). Aspirin attenuates spontaneous recurrent seizures in the chronically epileptic mice. Neurol. Res. 39, 744–757. 10.1080/01616412.2017.1326657 28481152

